# Editorial: Conceptualizing and measuring appetite self-regulation and its development in infancy and childhood

**DOI:** 10.3389/fnut.2022.976049

**Published:** 2022-08-17

**Authors:** Catherine G. Russell, Alan Russell

**Affiliations:** ^1^Institute for Physical Activity and Nutrition (IPAN), School of Exercise and Nutrition Sciences, Deakin University, Geelong, VIC, Australia; ^2^College of Education, Psychology and Social Work, Flinders University, Bedford Park, SA, Australia

**Keywords:** phenotypes, child, infant, appetite regulation, eating behavior, self-regulation, measurement, conceptualization

Effective self-regulation, including appetite self-regulation (ASR), is important for the healthy growth and development of children (1–3). One of the challenges facing researchers is identifying the theoretical basis for the measurement and conceptualization of ASR. To that end, parent report questionnaires and behavioral/observational measures have been developed and used to examine ASR and its development in childhood. These measures have typically been framed in terms of their relevance to outcomes such as weight gain and obesity rather than conceptually or theoretically.

Without a specific conceptual or theoretical foundation, a consequence is that the interpretation of results about ASR in childhood is often difficult. For example, while laboratory and questionnaire measures of eating in the absence of hunger (EAH) have been identified as predictive of weight in children, it is unclear whether children are more likely to eat in the absence of hunger due to increased attraction to food or to poor regulatory control. Similarly, while difficulty in food delay of gratification (DoG) tasks is linked to food intake and weight, it is uncertain whether this is due to the heightened attractiveness of the food or to poor regulatory control. Furthermore, without a clear theoretical foundation, construct definition, and measurement is more problematic.

The purpose of the Research Topic was therefore to contribute to advances in the conceptualization and measurement of ASR in infancy and childhood. One of the themes that emerged from the Research Topic collection centered around the bottom-up, top-down (dual process) theoretical model, where ASR is conceived in terms of bottom-up reactivity to food and hunger cues together with top-down regulatory control. Although using different measures, several authors drew on this framework to conceptualize ASR and its measurement.

The model was applied by Harris et al. who measured food responsiveness (pertaining to bottom-up reactivity) and temperamental regulation (pertaining to top-down regulatory control). They found that mothers used more food to soothe at 6 months for infants lower in regulation and higher in food responsiveness, that is, infants who displayed characteristics already suggestive of ASR difficulties. Stein et al. also used the bottom-up, top-down theory and added a distinction between general self-regulation, appetite regulation, and appetite self-regulation. They used a novel food delay of gratification task (pre-feed and mid-feed delay) with infants at 2, 8, and 16 weeks, with measures of infant distress and subsequent milk consumption. Components of general self-regulation, appetite regulation, and appetite self-regulation (especially the bottom-up food approach) were drawn on in the interpretation of the results. A significant aspect of this research is that it considers elements of emergent eating behavior regulation.

Reigh et al. describe a protocol for a study that will examine the relationship between biological, cognitive, and psychological factors and children's (4.5–6 years of age) ASR. In particular, they will investigate the influence of food form on intake in short-term energy compensation, which they argue is a proxy indicator of energy intake self-regulation. Overall, the research is informed by a dual (bottom-up, top-down) process model of ASR developed by the authors. They postulate several bottom-up and top-down influences and measures, some of which will be included in the research. The model incorporates food DoG, EAH, and energy compensation as components of ASR.

EAH was the focus of Hohman et al. Preschoolers from three classrooms completed both classroom and individual EAH tasks. The results suggested that EAH performs similarly in classroom and individual settings, indicating that the classroom protocol could be a viable alternative approach. The authors provide a helpful conceptual analysis of EAH processes, including possible increased bottom-up sensitivity and reactivity to food cues, and/or reduced top-down regulatory capacities together with a poorer ability to recognize internal satiety cues.

A second theme of the Research Topic was about relationships between questionnaires and behavioral or observational measures. Papaioannou et al. reviewed the evidence on this question and found that studies comparing questionnaire measures of ASR with other questionnaire measures showed the most evidence of significant associations, whereas studies comparing questionnaire measures with observational tasks mostly showed weak significant associations or none at all. Questionnaire measures seemed to be more associated with BMIz than behavioral measures. The results of their review raise fundamental questions about definitions of ASR-related constructs, their measurement, and their relationships. For instance, the authors note that the questionnaire measures are described as “traits”, whereas observational measures are more likely to be state-based or even as a measure of processes or skills (as the authors suggest could apply to the EAH protocol).

Consistent with the evidence from the Papaioannou et al. review, Hohman et al. found no relationships between EAH and parent-rated emotional overeating, enjoyment of food, and food responsiveness from the Children's Eating Behavior Questionnaire (CEBQ). They suggested that the CEBQ scales are about eating behaviors in general whereas the EAH measures behavior in a specific situation. Giuliani and Kelly also refer to the low convergence between survey and behavioral measures, in this case in relation to Executive Function (EF). These results in the Research Topic are similar to other findings about traits (questionnaire/self-report) vs. behavioral measures of self-control (4, 5). Papaiouannou et al. argue for more multi-method studies in recognition of the apparent multi-dimensional nature of ASR constructs in childhood.

Giuliani and Kelly contribute to questions about the conceptualization of DoG measures by investigating possible underlying processes in the food DoG task. They examined relationships between two food DoG tasks (snack delay and tongue task) and six cognitive measures that have been suggested to be implicated in top-down regulatory control (such as the Flanker task and Go/NoGo tasks). The cognitive measures were more consistently correlated with performance on the tongue task than the snack delay task. The authors raise the question of whether different DoG tasks could rely on separate underlying cognitive processes.

A third theme that emerged from the Research Topic was the contributions of variable-centered vs. person-centered approaches. As discussed by Russell et al., person-centered approaches can provide new insights and perspectives on ASR in childhood, especially in relation to fundamental processes and the components of ASR. This is achieved by identifying subgroups of children with different behavioral/psychological profiles on ASR and related measures, using latent class/latent profile analyses. For example, ASR difficulties in some children could arise from increased bottom-up reactivity to food cues, in other children from a limited top-down regulatory capacity, and in some children from a combination of bottom-up and top-down factors. Potential subgroups can be identified from cross-sectional data as well as from developmental trajectories. Person-centered approaches also facilitate analysis of the role of co-variates such as parent and family variables in ASR and its development. The importance of identifying subgroups in this way is founded partly on the evidence of very large individual differences in measures of ASR and trajectories of weight gain and obesity.

Russell et al. also applied a person-centered approach to describe appetitive trait trajectories across infancy and related those trajectories to infant and parent characteristics to understand emergent ASR. The authors used a group-based multi-trajectory analysis. Three multi-trajectory phenotype groups were identified. For example, the first group was described as food avoidant tending to a low food approach over time. The authors argued that the trajectories have their origins in both infant and parent characteristics as well as parent behavior and cognitions. They suggest that for some infants, difficulties in ASR emerge early in life.

Francis et al. also took a person-centered approach. They measured behavioral self-regulation (BSR) (e.g., teacher reports of inhibitory control and impulsivity) and ASR-related traits (parent reports of food approach and food avoidance traits). Latent profile analysis yielded four profiles, two described as discordant across BSR and ASR and two as concordant. For example, a concordant profile involved higher levels of BSR (e.g., higher inhibitory control and lower impulsivity) and ASR (e.g., higher food avoidance and lower food approach). Parents in the latter profile reported parenting practices with the highest levels of child control in feeding and the lowest levels of parental pressure to eat. These results show how a person-centered approach can yield insights into the processes and components of ASR in childhood as well as possible relationships with parenting practices (whether parent-to-child influence or child-to-parent influence).

Overall, the Research Topic supports a need for increased efforts to develop conceptual frameworks that will assist in constructing a definition and from there possibly even new approaches to measurement. The three main themes that emerged were around (i) the applicability of the top-down/bottom-up (dual process) model to understanding ASR, (ii) the limited convergence of questionnaire/self-report and behavioral/observational measures, and (iii) the value of both person-centered and variable-centered approaches to research. In proposing the original aims for the Research Topic, an area we noted was biological and neurological processes in ASR [e.g., (6–8)]. A limitation of the Research Topic is that, outside elements raised by Giuliani and Kelly, this aspect was not featured. This aside, insights gained into the conceptualization and measurement of ASR in childhood from the nine articles in this collection provide a basis for future scholarship on not only conceptualizing and measuring ASR but also the examination of influences on typical and atypical ASR development.

## Author contributions

CR and AR contributed equally to conceptualization and writing of the editorial. CR and AR contributed to the article and approved the submitted version.

## Conflict of interest

The authors declare that the research was conducted in the absence of any commercial or financial relationships that could be construed as a potential conflict of interest.

## Publisher's note

All claims expressed in this article are solely those of the authors and do not necessarily represent those of their affiliated organizations, or those of the publisher, the editors and the reviewers. Any product that may be evaluated in this article, or claim that may be made by its manufacturer, is not guaranteed or endorsed by the publisher.
